# Mental health impact of the Covid-19 pandemic on parents in high-risk, low income communities

**DOI:** 10.1177/0020764021991896

**Published:** 2022-05

**Authors:** Dana Alonzo, Marciana Popescu, Pinar Zubaroglu Ioannides

**Affiliations:** 1 Graduate School of Social Service, Fordham University, West Harrison, NY, USA; 2 Department of Social Work, Suleyman Demirel University, Isparta, Turkey

**Keywords:** Parental mental health, pandemic, low-income, marginalized communities, stress

## Abstract

**Background:**

COVID-19 has spread across the globe, resulting in significant changes in virtually every aspect of life. Mitigation efforts, like shelter-in-place orders, have taken a particular toll on parents who have had to navigate disruptions in work and/or school schedules. Research from high-income countries demonstrates increased parental anxiety, stress, depression, and burnout resulting from the pandemic. It is unclear if these outcomes are the same for parents in high-risk communities in low-income countries where pre-pandemic conditions were deleterious. This study addresses this gap and examines the mental health impact of the pandemic on parents in high-risk communities in Guatemala.

**Methods:**

A total of 330 individuals from 11 districts in Guatemala participated in the study and were assessed for sociodemographic characteristics and mental health impairment. Chi-squares were conducted for bivariate analysis. Multivariate analysis was conducted using binary logistic regression.

**Results:**

Bivariate analysis revealed differences between groups on burnout, with parents more often reporting feelings of burnout than nonparents (*p* < .001). Binary regression demonstrated that non-parents were 70% less likely to endorse feelings of stress as compared to parents (OR = .285; *p* = .014).

**Conclusion:**

Our findings underscore the importance of identifying the unique mental health impact of the COVID 19 pandemic on parents in high-risk communities. In high-risk communities, parental stress is a pressing problem that, if unaddressed, has the potential to result in even greater psychological distress and child maltreatment. Training community healthcare providers to assess and address parental stress can lead to increased community capacity and the development of a community-based network to serve as a first line of support for parents and their children.

## Background

The novel coronavirus (COVID-19) has spread across the globe, resulting in significant changes in virtually every aspect of daily life. National shelter-in-place orders, quarantines, and lock down restrictions have taken a particularly strong toll parents who have had to navigate drastic and often unanticipated changes in daily work and school routines for themselves and their children.

Since the onset of the pandemic in early 2020, almost all families have experienced a disruption in work and/or school schedules resulting from the pandemic and ensuing school, day care and afterschool program closures; the inability to work; job loss; and/or, shifts to remote learning and work (Gromada et al., 2020). For parents who were able to continue working (i.e. essential workers), juggling on-going work obligations with added childcare responsibilities and homeschooling has posed new and significant challenges. These challenges are further compounded by anxiety around virus exposure while at work and during travel to and from work. Parents who were able to work from home also face new challenges around establishing a new work routine and creating a workspace conducive to productivity while balancing child-care duties and homeschooling (Catalyst, 2020). Parents now at home unable to work or having lost their jobs found themselves having to manage childcare and homeschooling duties while struggling to address new financial concerns and economic stressors related to their inability to work and/or job loss (Conti, 2020; De Cao & Sandner, 2020).

During the best of times, parenting can be challenging. Concerns about behavior, health, education, socialization, and even everyday tasks, can serve as significant stressors for parents. When these ordinary stresses intensify they result in parental burnout, characterized as exhaustion in one’s parental role, emotionally distancing from one’s children, and loss of parental efficacy and sense of accomplishment (Roskam et al., 2018). Research has consistently demonstrated a link between parental burnout and increased rates of exhaustion, somatic complaints, poor quality sleep, a sense of incompetency as a parent (Mikolajczak et al., 2017, 2018; Roskam et al., 2017, 2018); feeling of being trapped in an uncomfortable situation with no way out (Hubert & Aujoulat, 2018); parental depression and anxiety, and child neglect and abuse (Cluver et al., 2020; Mikolajczak et al., 2017, 2018).

Added to conventional stressors of parenting, stressors caused by the threat or reality of parental unemployment, financial insecurity, low levels of social support, increased social isolation, lack of leisure time, lack of alone time characterizing pandemic-related conditions, can amplify normative stress and trigger significant psychological distress (Brooke et al., 2020; Parkes et al., 2015; Sorkkila & Aunola, 2020). Indeed, research examining mental health outcomes of parents during previous community-wide disasters supports this and demonstrates extensive negative impact of such experiences, particularly for families (Fussell et al., 2014; North, 2016). Additional research specifically points to heightened parental depression, stress and anxiety following communitywide disasters (Bolt et al., 2018; Kerns et al., 2013; Labarda et al., 2020; Maeda & Oe, 2017; Set et al., 2019).

More recently, research examining parental outcomes during the current COVID-19 pandemic demonstrates similar findings regarding negative mental health impact for parents, including increased anxiety, stress, depression, and burnout (Gromada et al., 2020). Research from the United States examining the mental health functioning of parents during the COVID 19 pandemic has demonstrated significantly more negative mental health outcomes for parents of young children compared to non-parents (Park et al., 2020; Pew Research Center, 2020; Russell et al., 2020). Further, research indicates that in the face of social distancing requirements and resulting unavailability of previous sources of social support and assistance, a concerning number of parents are experiencing burnout (Griffith, 2020). Additionally, a large nationally representative US survey found that parents of young children endorse significant stress, with 63% reporting that they feel they have lost their emotional supports during the pandemic (Center for Translational Neuroscience, 2020). Yet another large US survey found that 61% of parents of children between the ages of 5 and 7 reported heightened stress and feeling nervous, anxious or on edge (Gonzalez et al., 2020). Further still, another US study demonstrated that the number of stressors experienced due to the pandemic is associated with increased parental stress, anxiety, and depression (Brown et al., 2020). Another US study demonstrated worsening parental mental health functioning throughout the course of the pandemic, with 1 in 4 parents reporting increased psychological distress (Patrick et al., 2020). Lastly, another studied found that 1 in 4 quarantined parents report some negative mental health symptoms as compared to 1 in 20 non-quarantined parents (Brooks et al., 2020).

However, to date, research examining the mental health impact of the pandemic on parents in high-risk communities in low-income countries in scarce to non-existent, particularly in Latin American countries. Yet, across low-income countries, long-term civil conflict, poverty, food insecurity, stigma around mental illness, and a general lack of mental healthcare services contribute to increased maternal depression (Herba et al., 2016), parental mental health impairment (De Silva et al., 2007), and parenting difficulties (Parsons et al., 2012). Despite these pre-pandemic recognized challenges for parents and children in low-income countries, according to a joint World Bank and UNICEF study (Gentilini et al., 2020a), only 9 of 195 countries surveyed indicated implementing any childcare support initiatives in response to the current crisis. Latin-American countries’ response, while including different versions of cash transfers to offset the effects of the pandemic, do even less in regards to family support and child protection/child care (Gentilini et al., 2020b).

Given the pre-pandemic increased risk of mental health challenges among parents in low-income countries, it is reasonable to expect even greater impairment related to the onset of the pandemic than might be seen in high income countries, like the US. However, it remains unclear whether the mental health profile of parents generated by studies in the US and other high-income countries during the pandemic is relevant for/applicable to parents in under-served, often under-recognized and marginalized communities.

This study aimed to address this gap in current knowledge and increase our understanding of the mental health impact of the pandemic on parents residing in high-risk communities in Guatemala and discusses implications for parenting and childcare. In Guatemala, these marginalized communities are characterized by long-standing, high rates of poverty, pollution, over-crowding, violent crime and gang activity (i.e. extortion and narcotics trafficking). Domestic violence, child abuse and neglect, alcohol and substance abuse, and teen pregnancy occur at high rates. Access to electricity and water is limited, and Internet services are often lacking or unreliable when available. Given the pre-existing characteristics of these communities, it is likely that pandemic, which served to further limit already scarce resources, would only exacerbate existing mezzo and macro level stressors, resulting in significant psychological distress for parents.

Obtaining an accurate understanding of the unique mental health impact of the pandemic on parents from such marginalized, underserved populations is critical not only to inform targeted outreach, prevention, and intervention efforts for parents, but also to allow for the identification of children who may be in need of additional supports due to their parents impaired mental health functioning as research indicates that child outcomes related to community-wide disasters are worse among offspring of highly distressed caregivers and/caregivers who themselves experience negative mental health outcomes due to the disaster (Kerns et al., 2014; Kiliç et al., 2011; Masten & Narayan, 2012; Russell et al., 2020). This study can also contribute to identifying specific parenting challenges that could lead to child abuse or neglect or to an escalation of family violence, allowing for preventive measures to be integrated into intervention efforts.

## Method

### Sample

With the approval of the appropriate Institutional Review Board and in collaboration with Hunger Relief International and International Social Work Solutions, a total of 330 individuals from 11 high-risk districts in and around Guatemala City participated in a larger study, the Covid Care Calls (CCC) Program, and serve as the sample for the current study.

The CCC entailed semi-structured telephone interviews designed to elicit information regarding physical health, mental health, economic, and psychosocial status. Additionally, interviewers provided information, assistance, and appropriate referrals to vulnerable individuals at-risk of COVID-19 infection and experiencing other pandemic-related challenges. The objectives of the CCC program were to: (1) collect information regarding the extent and nature of physical health symptoms and psychological distress attributable to the pandemic; (2) make referrals for medical and mental health care; and, (3) prevent the spread of COVID-19 by providing education on evidence-based protective measures such as social distancing, regular handwashing, and mask-wearing. The study PIs designed the semi-structured interview, trained callers, and provided support and supervision to in-country staff. The calls are made by HRI-based social workers and psychology interns. Surveys were administered between June 6th, 2020 and September 30th.

### Measures

#### Sociodemographics characteristics

Through the semi-structured telephone survey, participants provided information regarding their sex, age, district of residence, number of children, and household composition.

#### Clinical characteristics

All symptoms of psychological distress were assessed using one main single item question with two subsequent follow-up questions asked of those who responded in the affirmative. Previous research indicates that single item measures of this type for assessing depression and other psychiatric symptoms have both adequate sensitivity and specificity to detect emotional distress in previous studies (Skoogh et al., 2010).

#### Anxiety

The lead question to assess for anxiety required participants to respond yes or no to the question, ‘Have you been feeling anxious since the pandemic began?’ If they answered yes, they were than asked two follow-up questions including, ‘On a scale of 1 to 10, 1 being the lowest and 10 being the highest, how anxious do you feel?’ and, to gather more information regarding nature and context of the anxiety, ‘Can you share with me the reasons for your anxiety?’.

#### Depression

The lead question to assess for depression required participants to respond yes or no to the question, ‘Have you been feeling depressed since the pandemic began?’ If they answered yes, they were than asked two follow-up questions including, ‘On a scale of 1 to 10, 1 being the lowest and 10 being the highest, how depressed do you feel?’ and, to gather more information regarding nature and context of the depression, ‘Can you share with me the reasons for your depression?’.

#### Stress

The lead question to assess for stress required participants to respond yes or no to the question, ‘Have you been feeling stressed since the pandemic began?’ If they answered yes, they were than asked two follow-up questions including, ‘On a scale of 1 to 10, 1 being the lowest and 10 being the highest, how stressed do you feel?’ and, to gather more information regarding nature and context of the stress, ‘Can you share with me the reasons for your stress?’.

#### Burnout/

The lead question to assess for burnout required participants to respond yes or no to the question, ‘Have you been feeling burned-out since the pandemic began?’ If they answered yes, they were than asked two follow-up questions including, ‘On a scale of 1 to 10, 1 being the lowest and 10 being the highest, how burned-out do you feel?’ and, to gather more information regarding nature and context of the burnout, ‘Can you share with me the reasons for your burnout?’.

#### Data analysis

The statistical analyses were performed using IBM SPSS Statistics for Windows, version 27 (IBM Corp., USA). Descriptive statistics (means, standard deviations, and percentages) were used to describe the sample demographic characteristics. Chi-squares were used to examine differences between parents and non-parents on sex and all mental health variables as all variables were dichotomized (mother/father; depressed/not depressed; anxious/not anxious; stressed/non stressed; burned-out/not burned-out).

Multivariate analysis was performed using binary logistic regression. Odds ratios were reported. Level of significance was set to *p* = .05. The variance explained by the mental health variables in distinguishing between parents and non-parents was also estimated using binary logistic regression and assessed with the Nagelkerke R Squared.

## Results

Table 1 presents the sociodemographic characteristics of the sample. Participants were on average 36.42 years old. They were largely female (66%) and mothers (68%). On average participants had X number of children.

**Table 1.** Sociodemographic characteristics of the sample.

Sociodemographic characteristic	N (%)	Mean (±SD)
Age (in years)		36.42 (±17.22)
Number of children		1(±1)
Sex		
Female	160 (66)	
Male	83 (34)	
Sex of parent		
Female	128 (68)	
Male	60 (32)	

Table 2 reports on the results of bivariate analysis. Parents and non-parents were not found to differ in terms of anxiety, stress, or depression. There were also no differences between the groups on sex. However, significant differences were found between the two groups on burnout, with parents more often endorsing feeling burned-out than non-parents (*p* < .001).

**Table 2.** Bivariate analysis of mental health symptoms of parents.

Mental health symptom	Parents (*n* = X)	Non-parents (n = X)	Chi-square	*df*	*p*-value
% (n)					
Parental sex					
Female	68 (128)	53 (19)	2.501	1	.114
Male	32 (60)	46 (16)			
Anxiety					
Yes	69 (140)	74 (34)	0.496	1	.481
No	31 (64)	26 (12)			
Stress					
Yes	56 (128)	59 (26)	0.718	1	.131
No	44 (100)	41 (18)			
Depression					
Yes	77 (179)	67 (35)	1.793	1	.181
No	23 (52)	31 (16)			
Burnout					
Yes	84 (234)	64 (32)	16.982	1	<.001
No	16 (46)	36 (18)			

Table 3 reports the results of the binary logistic egression. All mental health variables as well as sex of parent were retained in the regression model as previous research indicates significant differences between parents based on role (mother versus father) and on mental health functioning in these areas providing strong rationale to include them as variables of interest for this population.

**Table 3.** Binary logistic regression predicting parental mental health symptoms.

	OR	95% C.I.	*p*-value
Sex	0.651	0.250–1.516	.291
Depression	1.930	0.126–29.433	.636
Anxiety	0.410	0.141–1.191	.101
Burnout	3.231	0.197–52.953	.411
Stress	0.285	0.105–0.772	.014

Results of the regression indicate that non-parents were 70% less likely to endorse feelings of stress than parents (ORegression = egression.285; p < .001). No other variables were found to be significant.

## Discussion

This is a first study to measure the effects of COVID-19 on mental health particularly as it affects parents in low income, high-risk communities. The study aimed to explore the intersection between the pandemic and parental mental health and to inform the development of interventions to support this underserved, under-recognized population. Our findings underscore the importance of such intervention efforts to specifically target parental stress.

The reality of this pandemic with its widespread implications becomes even more evident through the results of this study. Affecting not only individuals, but families and entire communities, the pandemic places a higher burden on families with children. The findings indicate that the level stress for parents is significantly higher than for families and individuals without children. Although not unexpected, this particular finding suggests the need for contextualized interventions that will mitigate parental stress in the case of complex emergencies, for families with children (under the age of 18). This is especially important given the relationship between parental stress and child abuse, neglect, and family violence.

One interesting finding is that there was no significant difference between parents and non-parents on anxiety, burnout, or depression. This is in stark contrast to most US-based studies that identify an increase in anxiety, burnout, and/or depression for parents with young children as one of the immediate impacts of the pandemic (Griffith, 2020; Park et al., 2020; Pew Research Center, 2020; Russell et al., 2020) with implications for parenting and child care. One possible explanation of such difference could be linked to the complex emergency manifestation of the pandemic on communities dealing with compounded socioeconomic crises prior to the pandemic, thus normalizing severe deprivation and/or health concerns. In the absence of proper mechanisms or support services, people living in such communities might adapt their emotional responses by entering a generalized state of anxiety, or burnout; or by discounting of depressive symptoms as part of their daily lives.

Another somewhat surprising finding is that there were no significant differences based on parental roles, in regards to any mental health outcome, between mothers and fathers in the study. This is in contradiction with expectations regarding to caregiving roles in Latin American countries, which are disproportionately covered by women (Durand, 2011; Inter-American Commission of Women, 2020); and with the findings indicating that women’s levels of anxiety and depression in response to the pandemic’s impact on their parental role, and added caregiving responsibilities, are significantly higher than the rates of anxiety and depression in men (El Pais, 2020; Mayer, 2020). As such, we expected women to report greater levels of psychological distress related to their parenting responsibilities. One possible explanation could be the heightened sense of parental responsibility during the pandemic, and the extended time fathers end up spending with their children at home. While women tend to have disproportionately high caregiving responsibilities during these times, men’s roles shifted as well, and the presence of children at home in and of itself can elevate the level of stress father’s perceive, even if less involved/engaged in direct caregiving activities. Another possible explanation is that fathers’ sense of stress could be elevated by the changes of their role within the family (i.e. taking on activities and/or chores generally relegated to the mother), and the uncertainties they face in sustainably providing for their families in the face of job loss and/or insecure employment, and, health risks due to exposure while out trying to seek or maintain work.

Further considerations should be given to the elevated parental stress during the pandemic as a risk factor for child abuse and neglect, and family violence (De Cao & Sandner, 2020). In low-income communities, globally, in the context of ongoing crises, deprivation, lack of opportunities and support, childcare was not necessarily a stress-free or all-positive experience for children or parents even before the pandemic. According to a recent UNICEF study of 74 low and middle income countries, 80% of children ages 2 to 4 experienced some level of aggressive discipline, including either physical violence or emotional abuse from their main caregivers (parents) (UNICEF, 2020). In addition, even when positive parenting is the norm and the goal, under the additional stress created by the pandemic, parents’ wellbeing and ability to work towards providing best care for their children is significantly affected.

As the majority of study participants were women (66%) and the majority of parent respondents were mothers (68%) this study raises further concerns regarding gender-sensitive policies and protections provided to women in low-income, high risk communities. A lack of gender-inclusive social protection programs at national and regional levels, as well as the overrepresentation of women as caregivers, within a sociocultural context placing all caregiving responsibilities on mothers contribute to further escalations of stress levels for women. Prior to this pandemic, studies conducted in multiple countries found that 4 out of 10 women globally were outside social protection systems, rendering them fully exposed when the pandemic hit (UN, 2020). This has particular implications for communities like the ones in this study, where women need to balance childcare needs, care responsibilities for members of extended family, and securing the daily basic needs for their families; while social protection programs are limited, and the existing safety net mostly relies on informal networks of care.

### Limitations

There are a number of methodological limitations that should be acknowledged when considering our findings. First, the study did not employ a longitudinal design. As such, we are unable to determine if impairments in parental mental health functioning were maintained over time or if they improved or worsened throughout the course of the pandemic. Future research should examine the long-term mental health impact of the pandemic on parental mental health. Second, we focused on individuals residing in high-risk communities in a low-income country. Caution should be taken when generalizing the findings to high risk, low-income communities within middle and/or high-income countries as their mental health profile may differ. Lastly, this study did not explore the possible correlations between increased levels of stress, parenting, and safety and wellbeing. Due to the impact of the pandemic on family violence and child abuse (Bettinger-Lopez, 2020; SOS-Children’s Village, 2020) further explorations are needed.

## Conclusion

COVID-19 manifests itself as a complex emergency with immediate and long-term implications. The strict measures implemented to mitigate the spread of the virus have had concerning consequences on parents’ mental health. Within low-income high risk communities, as the findings of this study indicate, parents report significantly higher stress as a response to the pandemic, than nonparents. This response can be attributed to a number increased challenges related to caregiving in general and child care in particular compounded by potential job loss, risks of working outside the home, exposure to the virus, lack of access to an already limited support system, as well as an ongoing state of uncertainty as the pandemic is far from over.

While global responses to the pandemic aimed to address the higher level concerns related to controlling virus spread, little has been done to supplement support systems and strengthen the safety net for marginalized communities in low income countries, with Guatemala lagging behind, with no family support services or childcare programs added (Gentilini et al., 2020b). While policy recommendations that would include childcare supports outside the home might raise challenges related to the physical distancing requirements that serve as one of the core protective measures against COVID-19, other policies that address the barriers that prevent parents in these communities from accessing other forms of support (i.e. providing internet access to vulnerable and rural communities to support remote learning and activities) should be explored.

Further, interventions focused on increasing access of parents to informal and formal support networks and providing accurate information on existing services and how to access them could contribute to better assessment and response to mental health issues, while preventing escalation during times of complex emergencies. Training social workers and community health care workers to assess and address parental stress can lead to increased community capacity and the development of a community-based network to serve as a first line of support for parents and their children.
